# Vav1 GEF activity is required for T cell mediated allograft rejection

**DOI:** 10.1016/j.trim.2012.03.003

**Published:** 2012-06

**Authors:** Dirk Haubert, Jianping Li, Alexander Saveliev, Thomas Calzascia, Esther Sutter, Barbara Metzler, Daniel Kaiser, Victor L.J. Tybulewicz, Gisbert Weckbecker

**Affiliations:** a Novartis Institutes of BioMedical Research, Autoimmunity, Transplantation & Inflammation, 4002 Basel, Switzerland; b Division of Immune Cell Biology, MRC National Institute for Medical Research, London NW7 1AA, UK

**Keywords:** Vav, Allograft rejection, RhoGTPase, T cells

## Abstract

The GDP exchange factor (GEF) Vav1 is a central signal transducer downstream of the T cell receptor and has been identified as a key factor for T cell activation in the context of allograft rejection. Vav1 has been shown to transduce signals both dependent and independent of its GEF function. The most promising approach to disrupt Vav1 activity by pharmacological inhibition would be to target its GEF function. However, the contribution of Vav1 GEF activity for allogeneic T cell activation has not been clarified yet. To address this question, we used knock-in mice bearing a mutated Vav1 with disrupted GEF activity but intact GEF-independent functions. T cells from these mice showed strongly reduced proliferation and activation in response to allogeneic stimulation. Furthermore, lack of Vav1 GEF activity strongly abrogated the in vivo expansion of T cells in a systemic graft-versus-host model. In a cardiac transplantation model, mice with disrupted Vav1 GEF activity show prolonged allograft survival. These findings demonstrate a strong requirement for Vav1 GEF activity for allogeneic T cell activation and graft rejection suggesting that disruption of Vav1 GEF activity alone is sufficient to induce significant immunosuppression.

## Highlights

► The contribution of Vav1 GEF activity to allograft rejection was addressed. ► T cells with disrupted GEF activity showed reduced proliferation and activation. ► Mice with disrupted Vav1 GEF activity showed prolonged cardiac allograft survival. ► Vav1 GEF activity significantly contributes to T cell-mediated allograft rejection.

## 1 Introduction

Since the introduction of potent immunosuppressants such as calcineurin inhibitors and improved immunological matching, the risk of acute transplant rejection has been reduced considerably. However, despite a wide range of immunosuppressive agents, severe episodes of rejection still occur and chronic allograft rejection still poses a significant problem [1,2]. In view of both the nonimmune toxicities and the immunodeficiency complications caused by prolonged immunosuppression, there is a strong need to develop more specific immunosuppressive therapies [3,4].

T cells are central players in the process of transplant rejection and are involved both in the acute and chronic rejection phases, presenting an important target for immunosuppressive drugs. They drive graft rejection by direct and indirect mechanisms including apoptosis induction by cytotoxic T cells, cytokine release by T helper cells and by promoting T-dependent alloantibody responses [1]. Activation of allograft-specific T cells is induced by antigen presenting cells such as dendritic cells from both the donor and the host. Binding of MHC–allopeptide complexes to the T cell receptor together with concurrent costimulation triggers intracellular signal cascades leading to the activation and expansion of alloreactive T cells [5].

The members of the Vav family of guanine nucleotide exchange factors (GEFs) are central signaling molecules downstream of antigen receptors, and their deficiency severely affects antigen receptor signaling, lymphocyte development, activation and proliferation [6]. While Vav2 and Vav3 show a broad expression, Vav1 is primarily expressed in hematopoietic cells. Upon T cell receptor (TCR) engagement, Vav1 is phosphorylated and recruited to a TCR-proximal signaling complex including LAT, SLP76, GADS and phospholipase C γ1 (PLCγ1). Vav1 has been shown to integrate various different signal transduction pathways downstream of the TCR and costimulatory receptors leading to gene expression, cytoskeletal reorganization and proliferation [7]. Mice deficient for Vav1 show defects in thymic T cell development and activation of peripheral T cells [8]. T cells lacking Vav1 show reduced Ca^2+^flux, defective activation of extracellular signal-regulated kinase (ERK), Protein kinase C (PKC), the serine–threonine kinase Akt and T cell-APC conjugate formation [9–13].

Vav proteins contain a Dbl homology (DH) domain, which together with the adjacent plekstrin homology (PH) and C1 domains confers GEF activity toward the Rho-family GTPases Rac, Cdc42 and RhoA [14] [15]. In addition, they contain SH2 and SH3 domains which may mediate the GEF-independent functions of Vav. Phosphorylation of regulatory tyrosines in the acidic domain relieves the autoinhibitory interactions resulting in formation of the open, active conformation and activation of its GEF activity [16,17]. The relative contribution of the GEF-dependent and GEF-independent function of Vav1 for T cell signal transduction and activation still remains unclear. Conditional deletion of Rac1 and Rac2 resulted in a developmental block at the pre-TCR stage, resembling the phenotype of Vav1-deficient mice [18]. In addition, impaired T cell development in Vav1-deficient mice can be rescued by overexpression of constitutively active Rac1, indicating that Vav1 transduces pre-TCR signals via Rac1 [19]. The recent development of knock-in mice carrying a GEF-deficient Vav1 mutant (L334A, K335A, Vav1^AA/AA^) allowed to distinguish between GEF-dependent and -independent effects in primary cells for the first time. Analysis of these mice showed that the GEF activity of Vav1 is required for thymic development of T cells and some but not all signal transduction events like activation of Akt and integrin activation. Importantly, despite being dispensable for Ca^2+^ flux and ERK activation, the GEF activity of Vav1 is required for T cell activation and proliferation [20].

As a central player in T cell activation, Vav1 has been linked to several immune-mediated diseases including common variable immunodeficiency syndrome and multiple sclerosis [21,22]. We have previously shown an important role for Vav1 in alloreactive T cell responses and transplant rejection in a cardiac allograft transplantation model, demonstrating the immunosuppressive potential of Vav1 inhibition [23]. Targeting Vav1 activity by small molecules is difficult due to its several functions fulfilled by distinct domains. Blocking Vav1 adapter functions, which comprise multiple protein–protein interactions over large areas is difficult using small molecular weight inhibitors. Thus trying to disrupt the interactions between Vav1 and the downstream GTPases and hence its GEF function seems to be the more feasible approach. However, it is not clear if disruption of Vav1 GEF function alone is sufficient to induce immunosuppression. To address this question, we have used the GEF-deficient Vav1^AA/AA^ mice to analyze the contribution of Vav1 GEF function to allogeneic T cell activation and transplant rejection. We show that the GEF function is required for allogeneic T cell activation and proliferation both in vitro and in vivo. Vav1^AA/AA^ mice show prolonged allograft survival in the cardiac transplantation model indicating an important role for Vav1 GEF function in transplant rejection.

## 2 Material and methods

### 2.1 Mice

Mutant C57BL/6 mice carrying the GEF-inactivating mutation L334A/K335A in the *Vav1* gene (Vav1^AA/AA^) along with wild-type (WT) littermates have been described previously [20]. Animals were used between 8 and 12 weeks of age. Vav1^AA/AA^ or C57BL/6 WT female control mice were used as recipients of fully MHC-mismatched beige BALB/c (Charles River WIGA) primarily vascularized cardiac grafts. For the systemic graft-versus-host reactivity (GvH) model, female C.B-17 severe combined immune deficiency (SCID)-beige mice were supplied by Taconic, Bomholt Denmark and kept under specific pathogen-free (SPF) conditions. Mice were kept under conventional conditions in accordance with Swiss federal law and the NIH Principles of Laboratory Animal Care.

### 2.2 Antibodies and reagents

Fluorochrome-conjugated antibodies for FACS analysis against mouse CD4, CD8, CD25, IgM and IgG were purchased from BD Pharmingen and eBioscience. Antibodies for stimulation against CD3 (hamster anti-mouse CD3ε, 2C11) and CD28 (hamster anti-mouse CD28, 37.51) were obtained from BD Pharmingen.

### 2.3 T cell proliferation and activation

T cells from spleens and lymph nodes from Vav1^AA/AA^ and C57BL/6 WT mice were purified by negative selection using the EasySep T cell enrichment kit according to manufacturer's instructions (Stemcell Technologies, Vancouver, CA). Cells were labeled with 5 μM carboxyfluorescein diacetate succinimidyl ester (CFSE) for 10 min at 37 °C. 10^5^ cells were cultured in the absence or presence of plate-bound antibodies against CD3 and CD28 (1 μg/ml) for 72 h. Cells were stained with antibodies against CD4, CD8 and CD25 and analyzed by FACS in duplicates.

### 2.4 Mixed lymphocyte reaction (MLR)

T cells from spleens and lymph nodes from Vav1^AA/AA^ and C57BL/6 WT mice were purified as described for the T cell activation analysis. The one-way MLR was performed in 96-well plates using irradiated BALB/c splenocytes as allogeneic stimulators. Different numbers of purified responder T cells (1 × 10^5^, 2 × 10^5^, 4 × 10^5^) were mixed with different numbers of stimulator splenocytes (2 × 10^5^, 4 × 10^5^, 8 × 10^5^) and incubated for 4 days at 37 °C in a humidified incubator. After a 5 hour exposure to ^3^H thymidine, proliferation was measured in a Betaplate Counter (Wallac). Data are shown as mean values ± SD of triplicates.

### 2.5 Systemic graft-versus-host (GvH) model

Single cell suspensions were prepared from spleens of Vav1^AA/AA^ mice and WT littermate controls. After red blood cell lysis with ACK buffer (Sigma-Aldrich), cells were labeled with 2 μM carboxyfluorescein diacetate succinimidyl ester (CFSE) for 10 min at 37 °C. SCID-beige recipient mice were injected i.v. with 20 × 10^6^ unfractionated WT splenocytes or 40–60 × 10^6^ spleen cells from Vav1^AA/AA^ donors, respectively, to transfer 7 × 10^6^ T cells (as determined by anti-CD3 staining). Four days after transfer, cell suspensions were prepared from individual SCID recipient spleens and T-cell recovery was analyzed by four-color flow cytometry, CFSE, anti-CD4-PE, anti-CD8-PerCP and anti CD3-APC. Flow cytometry data were acquired on a FACScalibur (BD Biosciences) using CellQuest software. Data were analyzed with FlowJo software (Treestar, San Carlos, CA, USA). Estimates of CD4^+^ and CD8^+^ T-cell numbers per recipient spleen were calculated as the product of the total number of viable spleen cells (hemocytometer count, trypan blue exclusion) and the percentage of CD3^+^ CD4^+^ and CD3^+^ CD8^+^ spleen cells within the live lymphocyte forward/side scatter gate. The percentage of CD4^+^ or CD8^+^ T cells that had undergone a certain number of cell cycles was derived from marker settings on CFSE histograms. For cell cycle distribution plots, the arithmetic means and SD of all individual data per recipient group are shown.

### 2.6 Cardiac allotransplantation

Heterotopic heart transplantation was performed as described by [24] using aseptic surgery techniques. Briefly, animals were anesthetized using isoflurane. Following heparinization of the donor mouse, the chest was opened and the heart rapidly cooled with ice cold saline. The aorta and pulmonary artery were ligated and divided and the donor heart was stored in ice cold saline. The recipient was prepared by dissection and cross-clamping of the infra-renal abdominal aorta and vena cava. The graft was implanted with end-to-side anastomoses between the donor right brachiocephalic trunk and the recipient aorta and the donor right pulmonary artery to the recipient vena cava. Grafts were monitored by daily palpation and were considered rejected upon cessation of palpable ventricular contractions. Genotypes of all animals have been confirmed at the end of the study.

### 2.7 Histopathology and immunohistochemistry

Cardiac allografts and recipient hearts were cut transversally and fixed in 4% buffered formalin for histological evaluation. Fixed tissues were processed and embedded in paraffin according to standard procedures. Sections were stained with hematoxylin and eosin and Van Gieson for elastic fibers for light microscopic examination. Acute rejections were graded on scale 0 R (no rejection) to 3 R (severe acute cellular rejection) [25].

Cardiac allografts were analyzed immunohistochemically. Standard procedures were applied using mAbs anti-alpha smooth muscle actin (αSMA, clone 1A4, Dako-Cytomation, Glostrup, Denmark), anti-CD3 (clone CD3-12, Serotec Ltd, Oxford, UK), anti-CD45R (clone RA3-6B2, Serotec Ltd) and the streptavidin–biotin–peroxidase complex technique. Spleen tissue served as positive control sample. Negative immunohistochemical staining controls were obtained by replacing the primary antibodies with antibody isotype controls (Zymed Laboratories, Inc., San Francisco, CA, USA).

### 2.8 Alloantibody analysis

Purified CD4^+^ T cells from BALB/c spleens were incubated with serum from naive or transplanted wildtype or Vav1^AA/AA^ mice for 30 min on ice. Alloreactive antibodies were detected by FACS using FITC-conjugated anti-IgM and anti-IgG antibodies.

### 2.9 ELISA

Secreted levels of IL-2 in supernatants from stimulated cells were analyzed by ELISA according to manufacturer's instructions (DuoSet ELISA kit, R&D Systems, Minneapolis, MN, USA). Absorbance at 450 nm was measured using a SpectraMAX 190 ELISA reader (Molecular Devices).

### 2.10 Statistical analysis

Data were expressed as mean ± standard deviation (SD). Statistical significance was determined using a two-tailed, unpaired Student's T-test. (*p < 0.05, **p < 0.01, n.s. not significant). For the heart allograft transplantation model, significance was determined by Kaplan–Meier survival curves and Mantel–Cox test.

## 3 Results

### 3.1 Disruption of Vav1 GEF function affects T cell proliferation and activation

**Fig. 1:**
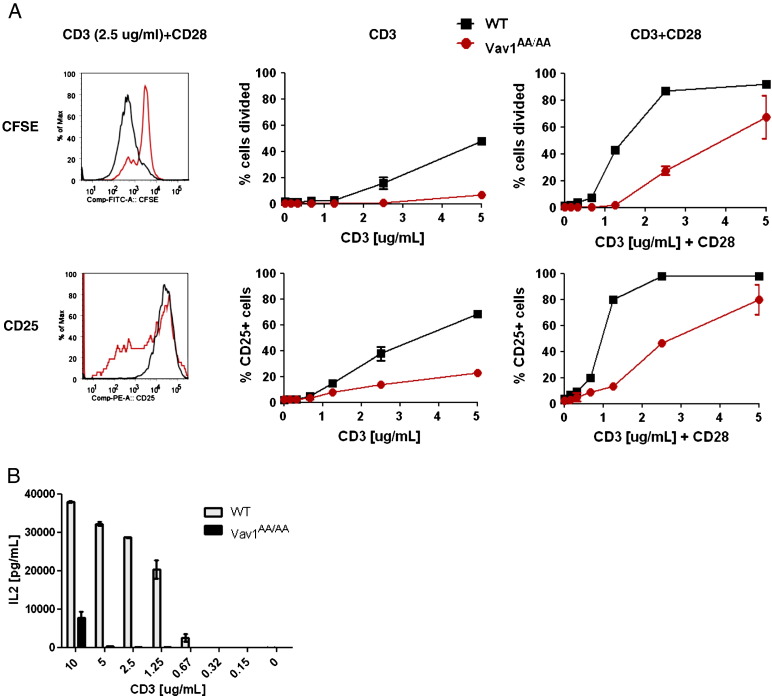
Vav1 GEF function affects T cell proliferation and activation. Purified T cells from WT or Vav1^AA/AA^ mice were seeded on plates coated with different concentrations of anti-CD3 antibody with or without soluble anti-CD28 antibody (1 μg/ml). After 72 h, percentage of cells that had divided at least once was measured by CFSE dilution, and activation was measured by staining for surface CD25 (A). IL-2 secretion in the supernatant of cells stimulated with anti-CD3 and anti-CD28 was measured by ELISA (B). Data are shown as mean ± standard deviation (SD).

To address the contribution of the GEF function of Vav1 for T cell activation in the context of allograft rejection, we made use of knock-in mice which carry a mutation in the DH domain of Vav1 (Vav1^AA/AA^). These mice express a mutated Vav1 which cannot activate Rac but has intact GEF-independent functions such as TCR-induced Ca^2+^ flux [20]. In order to determine if disruption of Vav1 GEF activity alone affects T cell proliferation and activation, purified T cells from Vav1^AA/AA^ and wild-type (WT) control mice were labeled with the fluorescent dye CFSE and stimulated on plates coated with antibodies against CD3 and CD28. After 3 days, proliferation and activation were assessed by flow cytometry. T cells from Vav1^AA/AA^ mice showed impaired proliferation compared to T cells from WT mice with both TCR stimulation and costimulation by CD28 (Fig. 1A), in line with previously published results [20]. Activation of Vav1^AA/AA^ T cells as measured by surface expression of CD25 was also impaired compared to WT T cells, although they reached almost WT levels of surface CD25 under strong stimulatory conditions. In addition, IL-2 secretion was severely reduced in Vav1^AA/AA^ T cells, which might contribute to the impaired proliferative potential (Fig. 1B).

**Fig. 2:**
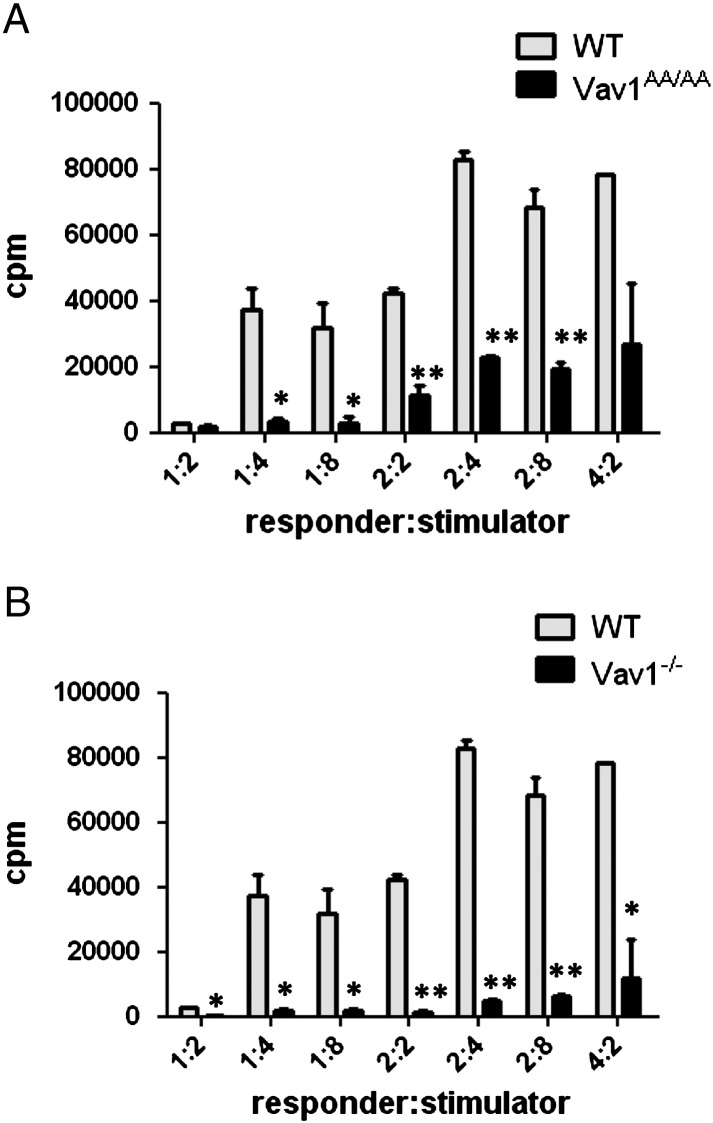
Allogeneic T cell proliferation is reduced in Vav1^AA/AA^ mice in a one-way mixed lymphocyte reaction. Different amounts of purified T cells from WT, Vav1^AA/AA^ (A) or Vav1^−/−^ (B) mice were co-cultured with fully MHC-mismatched irradiated BALB/c splenocytes for 4 days. Proliferation of allogeneic T cells was measured by thymidine incorporation. Values are shown as mean ± SD (*p < 0.05, **p < 0.01).

As T cell proliferation and activation by antibody-mediated stimulation is affected by the loss of Vav1 GEF activity, we wanted to know if Vav1 GEF activity also affects allogeneic T cell proliferation. To address this question, we cultured equal numbers of purified T cells of Vav1^AA/AA^ mice or WT control animals with irradiated splenocytes from fully mismatched allogeneic BALB/c mice in a one-way mixed lymphocyte reaction (MLR). Whereas WT T cells proliferated strongly in response to increasing numbers of stimulator cells, Vav1^AA/AA^ T cells showed a marked impairment of proliferation in response to allogeneic stimulation (Fig. 2A). To compare this phenotype to total Vav1 deficiency, we used T cells from Vav1^−/−^ mice in the MLR. T cells from Vav1^−/−^ mice also showed a strong proliferative defect as observed before (Fig. 2B) [23], which, despite the total Vav1 deficiency, is only slightly stronger compared to Vav1^AA/AA^ T cell. These results indicate that the GEF function of Vav1 has a key role in the proliferation and activation of allogeneic T cells.

### 3.2 Vav1 GEF activity is required for full allogeneic T cell expansion in vivo

**Fig. 3:**
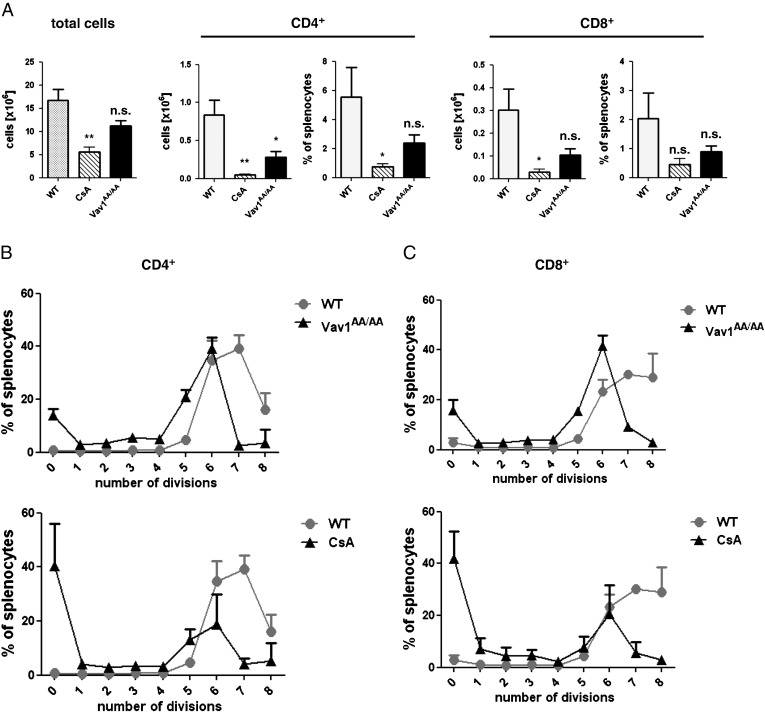
Vav1 GEF function affects allogeneic T cell proliferation in a systemic GvH model. CFSE-labeled donor splenocytes from WT or Vav1^AA/AA^ mice were injected into fully MHC-mismatched SCID/beige mice. As a control, additional mice receiving WT splenocytes were treated with CsA. Four days after transfer, proliferation of donor T cells was assessed by cell numbers and CFSE dilution. A, total cellularity in recipient spleens after transfer. B,C, proliferation of CD4^+^ (B) or CD8^+^ (C) T cells shown as percentage of cells which have undergone the indicated number of divisions (n = 4 per group). Values are shown as mean ± SD. Significance vs. wild-type values is shown (*p < 0.05, **p < 0.01).

To test whether the observed proliferation defect of Vav1^AA/AA^ T cells in vitro translates into an in vivo situation, we used splenocytes from Vav1^AA/AA^ or WT mice in a systemic graft-versus-host (GvH) model. CFSE-labeled splenocytes from Vav1^AA/AA^ or control mice were injected into BALB/c SCID mice, and alloantigen-driven proliferation of donor T cells in the recipient spleen was measured after 4 days. To account for the reduced number of single-positive T cells in Vav1^AA/AA^ mice which is caused by a developmental defect in the thymus [20], the number of injected splenocytes was increased accordingly to achieve equal number of injected T cells for the Vav1^AA/AA^ and WT groups. In addition, a third group treated with cyclosporine A (CsA) was included as a control for strong immunosuppression. In mice treated with CsA, the number of total splenocytes as well as CD4^+^ and CD8^+^ T cells in the spleen was reduced after 4 days compared to control mice. Interestingly, an almost equally pronounced reduction in splenocytes and T cells from Vav1^AA/AA^ mice was observed (Fig. 3A). To examine the proliferation of allogeneic T cells in more detail, the number of cell divisions was analyzed for CD4^+^ and CD8^+^ T cells by CFSE dilution. In contrast to the control mice, where most of the cells underwent 7 or more division cycles, around 18% of CD4^+^ T cells from Vav1^AA/AA^ donor mice did not divide at all, and fewer cells reached 8 division cycles (Fig. 3B). This impaired proliferation was also seen with CD8^+^ T cells, although not as pronounced. Treatment with CsA strongly affected proliferation, leading to more than 40% of CD4^+^ T cells that did not proliferate. Although T cells from Vav1^AA/AA^ mice showed an intermediate proliferative impairment compared to strong immunosuppressive conditions like CsA, these results suggest that Vav1 GEF activity is important for full allogeneic T cell expansion in the systemic GvH model.

### 3.3 Prolonged heart allograft survival in mice with disrupted Vav1 GEF activity

**Fig. 4:**
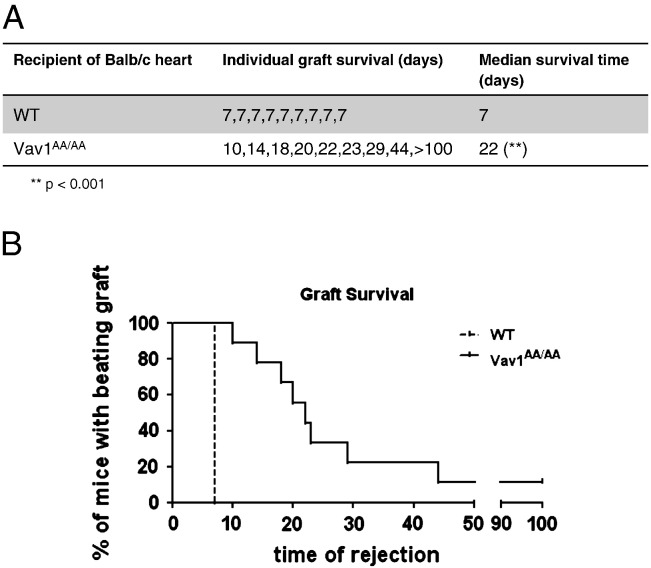
Vav1^AA/AA^ mice show prolonged allograft survival in a cardiac transplantation model. BALB/c cardiac allografts were transplanted onto WT or Vav1^AA/AA^ recipients in a C57BL/6 background. Graft beating was identified by palpation and used to measure graft survival. A, individual graft survival. B, percentage of mice with beating grafts shown as Kaplan–Meier survival curve.

We have observed that T cells with disrupted Vav1 GEF activity are impaired in allogeneic-driven proliferation and activation. To assess if this defect translates into an in vivo disease situation, we used WT and Vav1^AA/AA^ mice in a heart transplantation model. Allogeneic heart allografts from BALB/c donors were transplanted into WT or Vav1^AA/AA^ C57BL/6 recipients. All WT mice readily rejected the allograft after 7 days, whereas cardiac allograft survival in Vav1^AA/AA^ mice was significantly prolonged with a mean survival time (MST) of 22 days (Fig. 4). The majority of the animals rejected the allograft after 2–3 weeks, but two mice showed prolonged allograft protection of more than 3 months, with one animal reaching day 100 post-transplantation.

**Fig. 5:**
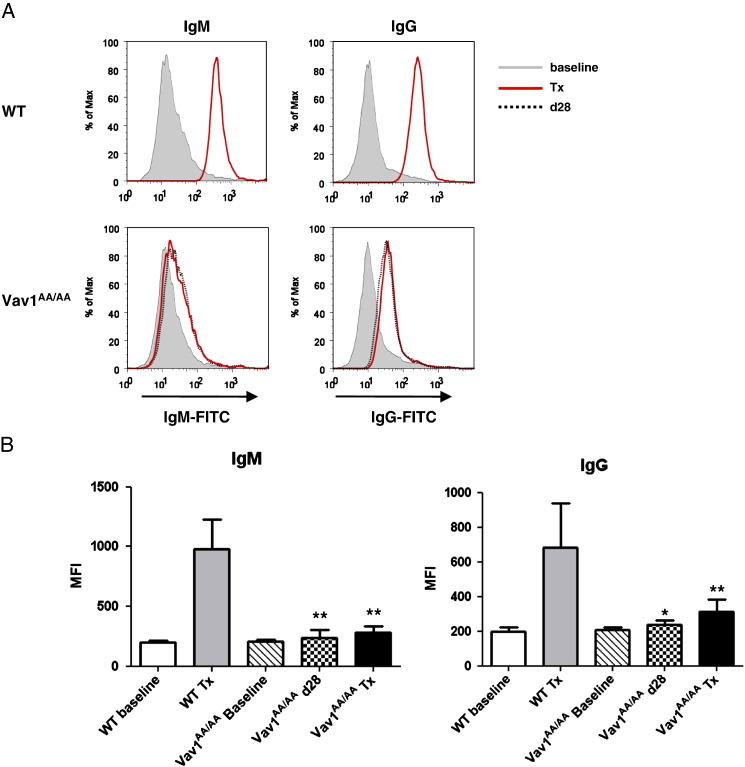
Reduced alloantibody response in Vav1^AA/AA^ mice. Serum from WT or Vav1^AA/AA^ mice was taken before transplantation (baseline), at the day of rejection (Tx) or during prolonged allograft survival at day 28 (Vav1^AA/AA^ only). Alloantibody levels against BALB/c alloantigens were determined by FACS and compared to baseline levels of naive mice. A, example of alloantibody levels in transplanted WT or Vav1^AA/AA^ mice at day 28 (dotted line), at day of rejection (solid line) compared to baseline levels (shaded). B, summary of antibody levels in all mice (n = 6 (Vav1^AA/AA^) n = 7 (WT)) at day of rejection (Tx) or at day 28 (d28). Data are shown as mean ± SD. Significance vs. WT Tx is shown (*p < 0.05, **p < 0.01).

Analysis of the alloantibody response against the graft showed a strong presence of IgM and IgG alloantibodies in transplanted WT animals at the day of rejection (Fig. 5). Vav1^AA/AA^ animals showed almost no increased alloantibody levels at the day of rejection, including those animals that showed only shortly prolonged graft survival. In addition, no alloantibody formation could be detected during the graft survival period at day 28, indicating that antibody-mediated rejection is severely compromised in Vav1^AA/AA^ mice.

### 3.4 Histopathology of cardiac allografts in mice with disrupted GEF-activity versus WT mice

In the WT mice the donor hearts showed acute cellular rejection (grade 3 R) with signs of endothelialitis present. Part of the donor hearts showed diffuse, severe myocardial necrosis, most likely ischemic and partially mixed with autolysis. No signs of rejection were found in syngeneic transplants.

**Fig. 6:**
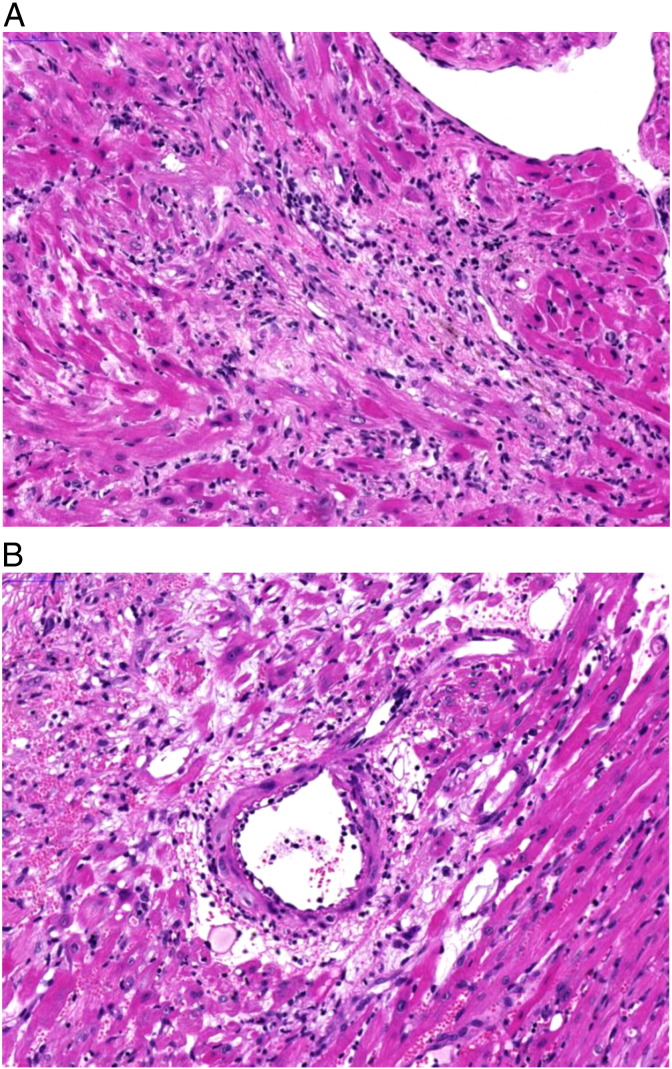
Histopathology of BALB/c cardiac allografts in Vav1^AA/AA^ C57BL/6 mice. A, graft showing interstitial fibrosis within the myocardium with mononuclear cellular infiltrates and mild hemorrhages (100 day survival). B, graft showing blood vessel with endothelialitis, perivascular mononuclear cellular infiltrates, hemorrhage and edema (44 day survival).

Cardiac allografts of Vav1^AA/AA^ mice also revealed areas of acute cellular rejection (grade 3 R) (Fig. 6). Myocardial necrosis was present but appeared not to be as diffuse as in WT mice. In contrast to WT mice, additionally multifocal areas of fibrosis were present in allografts transplanted into Vav1^AA/AA^ mice. This corresponds to a scattered progression to a chronic stage, which is supported by the observed prolonged allograft survival. Endothelialitis was present and single vessels showed a mild chronic vasculopathy. Immunohistochemical examination revealed interstitial cellular rejection composed of mainly T cells mixed with B cells, and a transplant vasculopathy with αSMA^+^ cells and T cells for both WT and Vav1^AA/AA^ mice (data not shown).

## 4 Discussion

Vav1 is a central molecule downstream of the TCR and has been recognized as a key mediator of T cell activation. We have previously shown that complete deficiency of Vav1 inhibits T cell activation leading to prolonged allograft survival in a transplantation model [23]. However, the relative contribution of the Vav1 GEF activity to its function in T cell activation in a disease setting has not been addressed. By using knock-in mice carrying a GEF-inactivating mutation in the Vav1 gene, we demonstrate that Vav1 GEF activity is essential for full T cell activation and proliferation by allogeneic stimulation. Disruption of only the GEF activity of Vav1, while leaving the adapter functions intact, leads to significantly prolonged allograft survival in a heart transplantation model. Our findings reveal a strong contribution of Vav1 GEF activity to allogeneic T cell activation, indicating that disruption of Vav1 GEF activity by therapeutic agents may be a novel way to induce immunosuppression.

Vav1 has been shown to participate in the activation of many signal transduction pathways downstream of the TCR, but which of these requires Vav1 GEF activity could only recently be addressed in primary cells [20]. It could be shown that some pathways such as TCR-induced Ca^2+^ flux and ERK activation are GEF-independent, whereas activation of others like the PI3K pathway requires Vav1 GEF activity. Still, T cell proliferation and activation after TCR stimulation are severely suppressed when Vav1 GEF activity is disrupted (Fig. 1) [20]. Only at very high concentrations of stimulating CD3 antibody and in the presence of costimulation by CD28, the requirement for Vav1 GEF activity is bypassed. Interestingly, proliferation and activation of T cells from Vav1^AA/AA^ mice are reduced to the same extent as of T cells from Vav1^−/−^ mice, indicating that Vav1 GEF activity is essential for this response [20]. A similar effect could be observed when T cells were stimulated by allogeneic splenocytes in a mixed lymphocyte reaction, where T cells derived from Vav1^AA/AA^ mice showed a strongly reduced proliferation almost as strong as T cells completely lacking Vav1 (Fig. 2). This is surprising, as T cells from Vav1^AA/AA^ mice have intact Ca^2+^ and ERK signaling, which is impaired in Vav1^−/−^ T cells [10,11]. However, one reason may be that the defects observed in Vav1^−/−^ T cells are only partial. Deficiency of all three Vav family members completely abolishes these signaling events, suggesting a redundant function for the other Vav proteins which could partially compensate Vav1 deficiency [26]. In addition, Vav1 has been shown to promote cell cycle progression via the PI3K pathway, which is defective in both T cells from Vav1^−/−^ and Vav1^AA/AA^ mice [20,27]. Furthermore, an important step in T cell activation, especially in the context of allogeneic stimulation, is the formation of the antigen presenting cell (APC)-T cell conjugate and downstream actin polymerization events. Vav1 transduces signals necessary for the activation of integrins important for APC–T cell conjugate formation, a function dependent on the GEF activity of Vav1 [12,13,20]. These pathways may explain the strong requirement for Vav1 GEF activity in allogeneic T cell activation.

T cells from Vav1^AA/AA^ mice also show a proliferative defect when injected into MHC-mismatched recipient animals in a mechanistic GvH mouse model (Fig. 3). The total number of Vav1^AA/AA^ T cells after 3 days was strongly reduced compared to WT T cells, and 18% of the cells did not divide at all. Interestingly, the majority of Vav1^AA/AA^ T cells reached 6 division cycles, showing that there was no complete block in proliferation. Rather, Vav1^AA/AA^ T cells seemed to have divided more slowly compared to WT T cells, which led to the reduced total numbers of cells. This is in contrast to T cells treated with the strong immunosuppressant CsA, where the majority of T cells did not divide at all. However, in a previous study, T cells from Vav1^−/−^ mice also did not show a complete block in proliferation but a similar delay in proliferation, which was enhanced in T cells from mice deficient in both Vav1 and Vav2 [23]. These findings suggest that disruption of Vav1 function only partially affects the TCR-induced proliferative signals which can be overcome by a stronger costimulatory environment in vivo. Vav1 GEF activity seems to be important for the Vav1-mediated proliferative response, as Vav1 GEF inactivation and total Vav1 deficiency have comparable effects. CsA, however, might affect Vav-independent TCR-induced signals and also different stimuli in addition to TCR engagement such as cytokines and costimulatory signals, which also contribute to T cell proliferation [28]. Furthermore, CsA has effects on other cell types and tissues resulting in strong general immunosuppression, which may explain the stronger response compared to Vav1 inactivation.

Vav1^AA/AA^ mice show prolonged cardiac allograft survival with a mean survival time of 22 days compared to WT animals which reject the allograft after 7 days (Fig. 4). These findings confirm the previously observed central role for Vav1 in allograft rejection [23]. Vav1^AA/AA^ as well as Vav1^−/−^ mice have reduced numbers of peripheral T cells due to a defect in thymic development [20], and we cannot exclude a partial effect of this reduction on allograft survival. However, Vav1^AA/AA^ T cells showed a strong defect in allogeneic T cell proliferation and activation in vitro and in vivo when equal numbers of T cells were used, indicating that the prolonged allograft survival in Vav1^AA/AA^ mice is likely to be caused by defective T cell activation. However, to fully confirm these findings, inducible genetic systems or specific Vav1 inhibitors will be needed.

Graft survival in Vav1^AA/AA^ mice is not as pronounced as in Vav1^−/−^ mice which lack the whole Vav1 protein, indicating that the GEF function of Vav1 affects only part of the processes mediating rejection [23]. This could also account for the high variation in allograft survival time observed for the Vav1^AA/AA^ mice. A similar effect has been observed in a study where disruption of Vav1-induced Rac activation by pharmacological interference resulted only in moderately prolonged cardiac allograft survival [29]. Histological examination showed signs of acute cellular rejection in the allografts of both WT and Vav1^AA/AA^ recipient mice, but enhanced fibrosis present in the Vav1^AA/AA^ allografts indicates progression to a more chronic stage of rejection compared to acutely rejected WT allografts (Fig. 6). This is in line with the observed histological features including acute cellular rejection and interstitial fibrosis for Vav1^−/−^ mice with an allograft survival time below 100 days [23].

Antibody-mediated rejection seems to require Vav1 GEF activity, as the formation of alloantibodies is almost absent in transplanted Vav1^AA/AA^ mice (Fig. 5). Antibody levels do not correlate with graft survival times in individual animals, suggesting that the variations in graft survival time are caused by different mechanisms. Vav1 has been implicated in T cell dependent antibody formation, and it would be interesting to see if the GEF function of Vav1 is required for general antibody responses [30,31]. Correct migration and localization of activated T cells to antigenic tissue are essential for developing an immune response. Vav1 has been implicated in SDF-1-dependent cell migration, and has been shown to be important for the retention of T cells at the sites of inflammation [32,33]. Vav1^−/−^ T cells fail to form sustained interactions with local APCs which reduce their ability to initiate a local immune response. Integrin-mediated adhesion and APC–T cell conjugate formation require Vav1 and its GEF activity, which may be a mechanism by which Vav1 GEF activity contributes to allograft rejection [20].

Costimulation is an important factor for allogeneic T cell activation, and blockade of costimulatory pathways has shown promising results in preventing transplant rejection [5]. Vav1 has been shown to link CD28 costimulation to T cell activation [34–36]. The GEF function of Vav1 could contribute to its role downstream of CD28, as Vav1 can enhance CD28-induced activation of transcription factors like NFκB via a Rac-dependent pathway [37]. In addition, CD3/CD28-induced proliferation and activation of T cells in vitro requires Vav1 GEF activity (Fig. 1) [20]. However, other costimulatory signals like ICOS, complement or OX40 contribute to T cell activation during graft rejection [5]. Whether Vav1 and its GEF function are involved in these different costimulatory signaling events has not been clarified yet. It is possible that Vav1 transmits different costimulatory signals independently of its GEF activity, which may partially account for the difference in graft survival between Vav1^−/−^ and Vav1^AA/AA^ mice. Analysis of the effector phenotype of Vav1^AA/AA^ T cells and their activation in vivo using TCR transgenic mice could also give more insights into the mechanisms of Vav1 GEF activity for allogeneic T cell responses.

## 5 Conclusion

In conclusion, we have demonstrated that the GEF activity of Vav1 is important for allogeneic T cell activation and proliferation. Disruption of Vav1 GEF activity in mice led to impaired alloreactivity and resulted in prolonged cardiac allograft survival. Our results show a significant contribution of Vav1 GEF activity to its role in T cell mediated rejection and indicate a potential novel way to induce immunosuppression by targeting Vav1 GEF activity.

## Authorship

DH performed research and wrote the paper; JP, TC, BM, ES, DK designed and performed research; VT and AS contributed mice and scientific input; GW initiated the concept and provided input to research and paper.

## Conflict of interest

The authors DH, JP, TC, BM, ES, DK and GW are employees of Novartis Pharma AG, Basel, Switzerland.

## Funding sources

All funding has been provided by Novartis Pharma AG, Basel, Switzerland.
